# Design and rationale of a randomized control trial testing the effectiveness of combined therapy with STAtin plus FENOfibrate and statin alone in non-diabetic, combined dyslipidemia patients with non-intervened intermediate coronary artery disease - STAFENO study

**DOI:** 10.1186/s13063-020-04291-5

**Published:** 2020-04-22

**Authors:** Taek-Geun Kwon, Albert Youngwoo Jang, Sang Wook Kim, Young Joon Hong, Jang-Ho Bae, Sung Yun Lee, Sang-Hyun Kim, Seung Hwan Han

**Affiliations:** 1 Division of Cardiology, Department of Internal Medicine, College of Medicine, Konyang University Hospital, Daejeon, Republic of Korea; 2 Division of Cardiology, Department of Internal Medicine, Gachon University College of Medicine, Gil Hospital, Incheon, Republic of Korea; 3 Division of Cardiology, Department of Internal Medicine, Cardiovascular-Arrhythmia Center, Chung-Ang University Hospital, Seoul, Republic of Korea; 4 Division of Cardiology, Department of Internal Medicine, Chonnam National University Hospital, Gwangju, Republic of Korea; 5 Division of Cardiology, Department of Internal Medicine, Inje University Ilsan Paik Hospital, Goyang, Republic of Korea; 6 Division of Cardiology, Department of Internal Medicine, Cardiovascular Center, Seoul National University College of Medicine Boramae Medical Center, Seoul, Republic of Korea

**Keywords:** Residual cardiovascular risk, Statin, Fenofibrate, Combination therapy, Virtual histology intravascular ultrasound, Randomized control trial

## Abstract

**Background:**

Despite the chronicled success of low-density lipoprotein cholesterol (LDLc)-lowering statin therapy, substantial residual cardiovascular (CV) disease risk remains a problem worldwide, highlighting the need to for combination therapies targeting non-LDLc factors, such as with fenofibrate.

**Methods/design:**

The STAFENO trial is a prospective, randomized, open-label, multi-center trial to compare the effect of statin plus fenofibrate with statin alone on the reduction and stabilization of plaque in non-diabetic, combined dyslipidemia patients with non-intervened, intermediate coronary artery disease (CAD) using virtual histology-intravascular ultrasound at 12 months. A total of 106 eligible patients are planned to be randomized to receive either a combination therapy (rosuvastatin 10 mg plus fenofibrate 160 mg/day) or monotherapy (rosuvastatin 10 mg/day) for 12 months. The primary endpoint of this study is the percentage change in the necrotic core volume. Secondary endpoints include changes in tissue characteristics and 1-year major CV events, including all-cause mortality, CV mortality, nonfatal myocardial infarction, stroke, and revascularization of the intervened and non-intervened lesions.

**Discussion:**

The STAFENO trial will address whether combination treatment of statin and fenofibrate has an additive beneficial effect compared to statin alone on the reduction and stabilization of plaque and CV events in non-diabetic, combined dyslipidemia patients with non-intervened intermediate CAD.

**Trial registration:**

ClinicalTrials.gov, NCT02232360. Registered 9 February 2014.

https://register.clinicaltrials.gov/prs/app/action/SelectProtocol?sid=S0004ULE&selectaction=Edit&uid=U00023SZ&ts=2&cx=juppd2

## Background

Coronary artery disease (CAD) is the leading cause of cardiovascular (CV) disease in developed countries, with approximately 1,350,000 patients presenting with acute coronary syndrome (ACS) annually in the United States alone [1]. Although percutaneous coronary intervention and optimal low-density lipoprotein cholesterol (LDLc)-lowering statin therapy have substantially reduced recurrent CV events in ACS, two-thirds of the CV disease risk remains [2–7]; this highlights the need to redirect the current CV reduction algorithms to focus beyond LDLc reduction and the use of statins [7, 8].

In the 2016 European guidelines for the management of dyslipidemias [9], non-high density lipoprotein cholesterol (non-HDLc) is indicated as a strong independent risk factor and should be a a secondary treatment target (class IIa, level of evidence B). In these guidelines, in high-risk patients with triglyceride (TG) more than 200 mg/dL despite statin treatment, fenofibrate may be considered for use in combination with statins (class IIa, level of evidence B). Although the FIELD and ACCORD trials did not demonstrate favorable efficacy of fenofibrate in diabetic patients, these studies showed that fenofibrate treatment reduced the CV event rate in patients with high TG or low HDLc levels [10, 11]. A meta-analysis of fibrates on CV outcomes also confirmed a significant reduction in CV disease risk without an increase in drug-related adverse events [12]. These data suggest that statin and fenofibrate combination therapy is a potential alternative to statin alone in CAD patients with high levels of non-HDLc. Angiographically, mild atherosclerotic plaques account for 50% of recurrent CV events, further bringing non-culprit vessels into focus as a useful surrogate marker for future CV events [5]. Virtual histology (VH)-intravascular ultrasound (IVUS) allows the tissue characterization and quantitative assessment of plaque, which provides an extensive understanding of the non-culprit lesions [13]. Here, we seek to determine the additive impact of fenofibrate versus optimal statin therapy on non-culprit lesions in non-diabetic, combined dyslipidemia patients with non-intervened intermediate coronary artery disease using well-defined VH-IVUS criteria.

## Methods/design

### Study design and subjects

This is a prospective, open-label, randomized, multi-center trial to investigate the efficacy of statin plus fenofibrate treatment compared to treatment with statin alone in non-diabetic, combined dyslipidemia patients with non-intervened intermediate CAD.

A brief flowchart of the study is summarized in Fig. 1.

**Figure:**
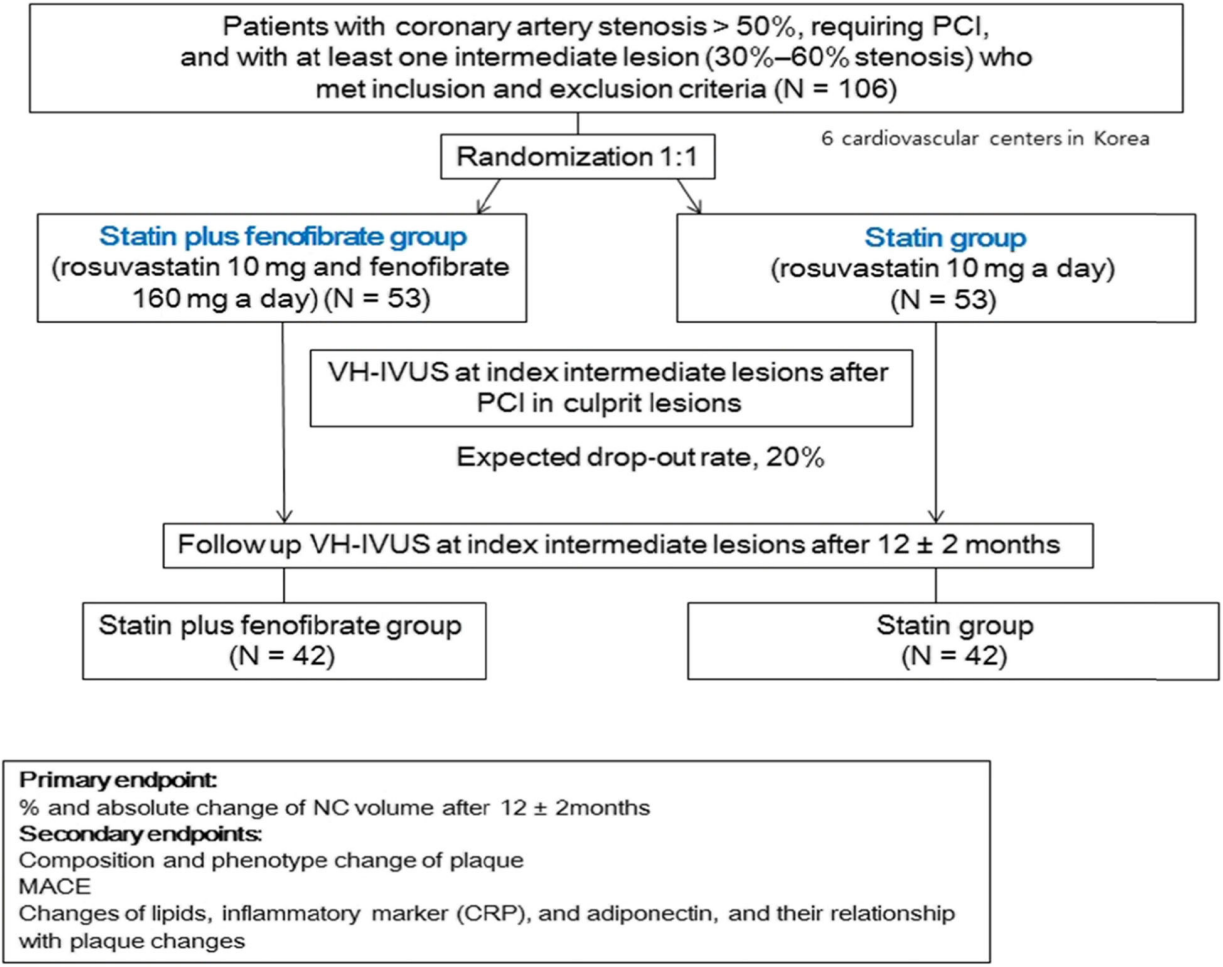
**Fig. 1** A Brief Flowchart of the Study

In non-diabetic patients with suspected CAD, coronary angiography (CAG) will be performed with the standard technique. Percutaneous coronary intervention (PCI) will be performed in culprit lesions based on the decision of an interventionist in participating centers within Korea [14]. Non-culprit lesions (index lesions) with intermediate stenosis (percentage diameter stenosis, 30–60%) by CAG will be evaluated by VH-IVUS if the patients meet the study inclusion and exclusion criteria. Data will be stored for off-line analysis.

Patients will be randomized at ratio of 1:1 using a random table (made by matches of age, sex, presentation of coronary artery disease and sequentially numbered) to receive either rosuvastatin 10 mg plus fenofibrate 160 mg or rosuvastatin 10 mg alone. Study drugs will be administered after the index VH-IVUS. Biochemical laboratory tests (fasting state) will be performed at the time of CAG and followed up at the third month (±2 months) and 12th month (±2 months). Follow-up CAG and VH-IVUS examination will be performed at the 12th month (±2 months). Clinical events will be followed up by office visit or by telephone contact if necessary. Adherence to the study drugs will be checked at every outpatient visit, and the decision to discontinue any of the study drugs will be discussed and checked under the recommendation of the responsible cardiologist. Other drugs which influence lipid profiles will be prohibited during the trial period.

### Study objectives and hypotheses

The primary objective of this study is to compare the changes in percentage necrotic core volume (NCV) and absolute NCV in non-culprit intermediate lesions in non-diabetic, combined dyslipidemia patients after 12 months’ therapy with statin plus fenofibrate compared to statin alone. The working hypothesis of this trial is that statin plus fenofibrate combination therapy is superior to statin alone therapy in reducing the 12-month percentage NCV and the absolute NCV. The secondary objective is to compare changes in percentage and absolute dense calcium (DC), fibrofatty (FF) and fibrous (F) plaques, the incidence of and changes in thin cap fibroatheroma (TCFA), the changes in variables of grey scale IVUS (volumes of external elastic membrane, lumen, and plaque), and the remodeling index between the statin plus fenofibrate and statin alone groups. In addition, secondary endpoints will include comparisons of major adverse cardiac events (MACEs) at 12 months, defined as all-cause death, CV death, nonfatal myocardial infarction (MI), stroke, and ischemia-driven revascularization of intervened and non-intervened lesions between the two groups. In order to identify the mechanisms of plaque stability and regression, the relationships between the variables of the VH-IVUS parameters and laboratory markers, including lipids, inflammatory markers, and metabolic markers, in the statin plus fenofibrate and statin alone groups will be investigated. The incidence of drug adverse effects will be another secondary endpoint of the study.

### Study organization

A total of six CV centers in Korea will participate in this trial; these centers include Gachon University Gil Hospital, Chung-Ang University Hospital, Chonnam National University Hospital, Konyang University Hospital, Seoul National University Borame Hospital, and Inje University Ilsan Paik Hospital. The protocol of the trial is registered at http://clnicaltrials.gov (NCT02232360).

This study is an investigator-initiated study sponsored by Dae-woong pharmaceutical company (Seoul, Korea, + 82,025,508,800). The authors alone are responsible for the design and execution of the trial, related statistical analyses, all aspects of the manuscript preparation, including the drafting, editing, and final content, data monitoring, and reporting of adverse events to the institutional review board (IRB) of each participating center.

### Study population and entry criteria

Inclusion criteria are as follows: (1) age ≥ 20 years; (2) presence of non-culprit intermediate lesions confirmed by index CAG; and (3) combined dyslipidemia (baseline LDLc ≥ 70 mg/dL and non-HDLc ≥ 130 mg/dL in statin-naïve patients, and baseline LDLc ≤ 100 mg/dL and non-HDLc ≥ 100 mg/dL in patients on statin treatment). A non-culprit lesion is defined as a VH-IVUS-feasible native coronary lesion with 30–60% stenosis, 2.0–4.0 mm in diameter by visual estimation during CAG, and located more than 10 mm apart from the intervened lesion.

Exclusion criteria are as follows: (1) diabetic patients; (2) poor cardiac, renal, or hepatic function; (3) explicit side effects and/or contraindications to lipid-lowering agents, including pregnancy, breastfeeding, and familial hypercholesterolemia; (4) very high triglyceridemia (TG ≥ 500 mg/dL); (5) lesions that might cause difficulties for VH-IVUS. Detailed inclusion and exclusion criteria are summarized in Table 1.

**Table 1:** Inclusion and exclusion criteria

**Inclusion criteria**	
• Patients with coronary artery disease who were 20 years of age or older and required coronary angiography	
• Patients who require PCI in culprit lesions	
• Intermediate coronary artery stenosis (diameter stenosis ≥ 30% to ≤ 60% by visual estimation, diameter ≥ 2.0 mm to ≤ 4.0 mm, de novo lesion in native coronary artery, more than 10 mm distance from PCI sites) in which virtual histology-intravascular ultrasound (VH-IVUS) is feasible	
• Statin naïve subjects: Combined dyslipidemia (LDLc ≥ 70 mg/dL and non-HDLc ≥ 130 mg/dL)	
• In statin subjects: LDLc ≤ 100 mg and non-HDLc ≥ 100 mg/dL	
• Patients who provided written informed consent	
**Exclusion criteria**	
• Diabetic patients	
• Patients with a history of the use of other lipid-modifying agents (except statins) within the 2 weeks prior to the trial beginning	
• Cardiogenic shock	
• Heart failure with symptoms of New York Heart Association class III/IV or a left ventricular ejection fraction < 35%	
• Renal dysfunction (creatinine level ≥ 1.7 mg/dL) or dependence of dialysis	
• Hepatic dysfunction (transaminase level more than three times that of the normal limit)	
• Pregnancy or breastfeeding women or women of childbearing age	
• Familial hypercholesterolemia	
• Hypertriglyceridemia (triglyceride level > 500 mg/dL)	
• Lesions that might cause difficulties for VH-IVUS due to the following reasons: heavy calcification (> 90° arc), tortuous vessel with severe angulation, total occlusion, or major bifurcation lesions (side branch diameter > 2 mm)	
• Inability to take adequate antiplatelet therapy (aspirin, clopidogrel, ticagrelor, or prasugrel)	
• Thrombocytopenia (platelet count < 70 × 10^9^/L)	
• History of significant arrhythmia including ventricular tachyarrhythmia	
• Familial hypercholesterolemia	

### Randomization, interventions, and data assessment

**Fig. 2:**
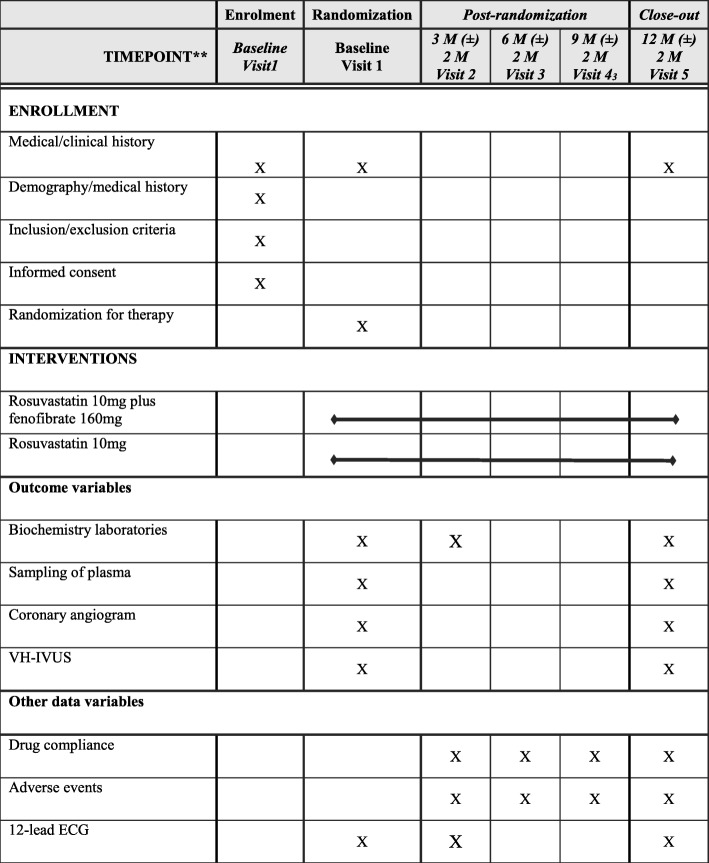
Schedule of enrolment, interventions, and assessments

The schedule of enrolment, interventions, and assessments is outlined in Fig. 2.

Written informed consent for subjects who meet clinical and angiographic inclusion and exclusion criteria will be obtained by an investigator. Personal information is planned to be corrected by interview and chart review. All personal information will be protected by an encoding process. Potential participants are informed of the purpose of the study, the potential hazards and risks, the potential benefits, the procedure, and the expected follow-up after intervention. They are informed that participation is voluntary and that withdrawal from the study may occur at any time. On the consent form, participants will be asked if they agree to the use of their data should they choose to withdraw from the trial. Participants will also be asked for permission for the research team to share relevant data with people from the universities taking part in the research or from regulatory authorities, where relevant. This trial involves collecting biological specimens for storage. Additional permission for storage and measurement of biological specimens will be acquired from participants according to the local IRB’s requests.

After the subjects are enrolled in the present study, subjects will be randomized to two groups using a random box table made by stratified randomization according to subjects’ age, sex, and clinical presentation (acute coronary syndrome or not).

### Research materials

#### VH-IVUS and quantitative coronary angiography

VH-IVUS using a synthetic-aperture-array, 20-MHz, 3.2-French catheter (Eagle Eye, In-Vision gold, Volcano Corp, rancho Cordova, CA, USA) with motorized catheter pullback (0.5 mm per second) after intracoronary administration of nitroglycerin 0.2 mg will be performed in non-culprit lesions with intermediate stenosis. During pullback, grayscale IVUS is recorded and raw radiofrequency data are captured at the top of the R wave for the reconstruction of the color-coded map by a VH-IVUS data recorder (Volcano Therapeutics). Enrolled lesions will be native vessels and should be located more than 10 mm apart from the intervened lesion. Cases where the patients’ condition is unstable or the characteristics of the lesions make it difficult to perform VH-IVUS in terms of severe angulated lesions, calcified lesions, or thrombus containing lesions, will be excluded.

Data from the VH-IVUS and CAG will be measured by an independent investigator who is blinded to patient group and characteristics. Data from VH-IVUS, such as percentage and absolute NCV, DC volume, F volume, FF volume, and presence of thin cap fibroatheroma (TCFA), and data for the gray IVUS, such as external elastic membrane, lumen, plaque volume, and the remodeling index, will be measured in the entire index segment and the most severe 10 mm segment centered on the minimal lumen area at baseline and after 12 months.

Data from CAG, such as reference vessel diameter, minimal lumen diameter, and lesion lengths, will be measured by a dedicated quantitative coronary angiography (QCA) program.

#### Laboratory assessment

Lipid profiles (total cholesterol, HDLc, TG, apolipoprotein A-1, and apolipoprotein-B), hs CRP, metabolic markers (fasting blood sugar and hemoglobin A1c), renal and hepatic function, and creatine phosphokinase (CPK) will be measured at baseline and after 12 months. At both baseline and after 12 months, sampled blood will be collected in ethylenediamine tetraacetic acid (EDTA) bottles, centrifuged for 15 min at 1000 g, and stored in a freezer at temperatures below − 70 °C. Using stored plasma, the levels of adiponectin, lipoprotein-associated phospholipase A2, and fatty acid binding protein will be measured at the end of the study using specific ELISA kits.

#### Study endpoints

The primary endpoint of this study is the comparison of the change in percentage NCV and absolute NCV between statin plus fenofibrate and statin alone groups of non-diabetic, combined dyslipidemia patients with non-intervened intermediate coronary artery disease at 12 months. The secondary endpoints are as follows: 1) comparisons of the changes in other tissue components of percentage and absolute plaque volume in index non-intervened lesions, including DC, FF, and F; 2) change in plaque phenotypes such as TCFA; 3) change in grey IVUS parameters, including vessel area/volume, lumen area/volume, plaque area/volume, and remodeling index; 4) MACEs for 12 months, including all cause death, CV death, nonfatal myocardial infarction, stroke, and ischemia-driven revascularization of intervened and non-intervened lesions; 5) relationships between the changes in tissue components of plaque and changes in laboratory findings; 6) adverse reactions to the study drugs, including myalgia, elevation of CPK more than twice the upper limit of normal, and hepatic dysfunction (alanine aminotransferase more than three times the upper limit of normal).

#### Participant follow-up

Clinical follow-up will take place at 3, 6, and 9 months and 1 year by clinical visit or telephone interview if needed. Laboratory follow-up will take place at 3 months (not obligatory) and 1 year. Follow up CAG and VH-IVUS will take place 12 months after randomization. At all planned contacts during the treatment period, patients will be asked whether they have adhered to the correct drug administration. Subjects with trial-related adverse events during the study period will be compensated for the standard of care by LIG Insurance Co., Ltd (Seoul, Korea).

## Statistical considerations

### Sample size calculations

Based on a previous study [15], a sample size of approximately 42 patients per treatment group was calculated to provide 80% power (assuming a standard deviation of 7.82) to detect a difference of 4.78 in percentage NCV and to detect the superiority of statin plus fenofibrate compared to statin alone with a two-sided alpha level of 0.05. With a statin plus fenofibrate:satin alone sampling ratio of 1:1 and a dropout rate of 20%, a final sample size of 53 patients per treatment group (total 106 patients) was specified in order to provide an adequate number of evaluable patients. To the best of the researchers’ knowledge, no study has been conducted on the change of tissue characteristics after fenofibrate treatment. We hypothesized that fenofibrate has an effect on percentage NCV as much as that of 10 mg atorvastatin [15]. In addition, a previous study [16] consisting of 100 patients with non-significant lesions demonstrated a significant decrease in NCV in the rosuvastatin (10 mg/day) treated group but not in the simvastatin treated group (20 mg/day).

### Statistical analyses

The primary endpoint of percentage NCV and absolute NCV will be analyzed using a paired-sample *t*-test or Wilcoxon rank-sum test for changes within each group, and an unpaired *t*-test or Mann–Whitney U test for differences between the two groups. Continuous variables will be expressed as mean ± standard deviation and compared by either a Student’s *t*-test or Mann–Whitney U test based on whether or not the data are normally distributed. Categorical variables will be expressed as number or percentage and compared by χ^2^ or Fisher’s exact test. Pearson correlation test will be used to evaluate the correlation between the changes in percentage NCV and absolute NCV, and changes in laboratory findings from baseline to follow-up. These statistical analyses will be performed in the entire index non-culprit segment and the most severe 10-mm segment centered on the minimal lumen area.

Multivariable linear regression analysis will be performed in order to identify the independent predictors of the reduction in percentage NCV. To analyze the secondary endpoints, VH-IVUS parameters, grey IVUS parameters, and angiographic parameters will be analyzed using a paired sample *t*-test or Wilcoxon rank-sum test for changes within each group, and an unpaired *t*-test or Mann–Whitney U test for differences between the two groups. Correlations between these parameters and laboratory variables will be evaluated by the Pearson correlation test.

Changes in the phenotypes of the index lesions between the two groups will be compared by χ^2^ or Fisher’s exact test. The composite endpoint of CV events will be analyzed by comparing the Kaplan–Meier event rates using a log-rank test. A *p* value < 0.05 will be considered statistically significant.

## Trial organization

### Executive Committee

The Executive Committee will be composed of the study principal investigator and the sub-principal investigator of each participating center. This committee will approve important protocol modifications and the final trial design. Protocol modifications will be issued to the Data Safety Monitoring Board (DSMB) after communicating with local IRBs. The Executive Committee will also be responsible for the final results and determining the methods of presentation and publication.

#### Endpoint Adjudication Committee

The Endpoint Adjudication Committee (EAC) comprises interventional and non-interventional cardiologists who do not participate in the study. This will be in charge of the development of specific criteria of endpoints based on study protocol and will review the accuracy and adequacy of reported endpoints.

#### Data Safety Monitoring Board

The DSMB is composed of general and interventional cardiologists. The DSMB policies will be in accordance with relevant regulatory guidelines. The board members will be independent of the trial. The safety data from the current study will be reviewed by the DSMB committee. Recommendations will be made based on safety analyses of unanticipated serious adverse events and protocol deviation. The DSMB will be provided with all cumulative safety data throughout enrollment and follow-up periods to ensure patient safety. All DSMB reports will be confidential, but will be available to relevant agencies upon request.

#### Ethics approval and auditing

The study is performed according to the principles of the Declaration of Helsinki and the common guidelines for clinical trials (ICH-GCP). This study has been approved by the IRB of Gachon University Gil Hospital and each participating center. Auditing will be carried out by the IRB of each participating center for adequacy of subjects enrollment, intervention, and data monitoring.

## Discussion

The STAFENO trial will determine the efficacy of fenofibrate in the reduction and stabilization of plaque in index non-culprit intermediate lesions with background treatment of statin in non-diabetic patients with mixed dyslipidemia. In addition, plausible mechanisms of plaque changes will be investigated through the direct relationships with changes in lipids, inflammatory markers, and metabolic markers. The STAFENO trial will provide insights into the combined treatment with statin and fenofibrate in non-diabetic, intermediate CAD patients with combined dyslipidemia.

### Rationale behind selecting fenofibrate for residual risk reduction

Despite the chronicled success of LDLc-lowering statin therapy, residual CV disease risk remains a worldwide burden, highlighting the need for targets other than LDLc, such as niacin and fibrates [7, 8, 17]. Furthermore, the prevalence of hypertriglyceridemia is greater than hypercholesterolemia within the Korean population [18–20], in the Chinese National Survey [21], and in Japanese men (http://www.mhlw.go.jp/bunya/kenkou/eiyou/h25-houkoku.html). In addition, a 2007 Korean National Survey revealed that 33.2% of the general population had hypertriglyceridemia (triglycerides ≥ 150 mg/dL), and that 50.2% of them had low HDLc levels (men < 40 mg/dL, women < 50 mg/dL) [22]. It is important to mention that recent genetic studies and randomized trials demonstrated that low HDLc might not be a cause of CV disease; instead, the risk of CV disease may be exacerbated by hypertriglyceridemia and remnant cholesterol [7, 23]. Therefore, the treatment of combined dyslipidemia is very important in reducing remnant CV disease risk, especially in the Asian population [7]. However, for the treatment of combined dyslipidemia, in terms of high LDLc and hypertriglyceridemia, or high non-HDLc, niacin plus statin combination therapy was associated with an increased risk of adverse effects, such as new-onset diabetes mellitus, diabetic complications, gastrointestinal problems, bleeding, and infection, and a lack of improvement in clinical outcomes, thereby limiting its use [24, 25].

Fibric acid, a synthetic ligand of the peroxisome proliferator-activated receptor (PPAR) α, is an effective drug for lowering plasma TG and increasing HDLc [8, 26]. In addition, PPARα activation by fenofibrate improves insulin sensitivity and endothelial function and decreases thrombosis and vascular inflammation [27, 28]. In ApoE*3 leiden mice fed a high cholesterol diet, fenofibrate reduces atherosclerosis more than can be explained by the lowered total plasma cholesterol. The anti-atherogenic effects of fenofibrate might be responsible for the regression of coronary plaque [29] and reducing the coronary events associated with atherosclerosis. Furthermore, impaired recruitment of monocytes/macrophages, reduced vascular and systemic inflammation, and stimulation of cholesterol efflux may all contribute to these beneficial effects of fenofibrate [30].

Our group has previously shown that in humans PPARα activation by fenofibrate has additive beneficial effects, improving insulin sensitivity and endothelial function, while decreasing thrombosis and vascular inflammation compared to treatment with statin alone [27, 28]. Although the FIELD and ACCORD trials in diabetic patients did not demonstrate a significant reduction in the primary composite outcome of the addition of fenofibrate, post hoc analysis has shown that the use of fenofibrate in patients with elevated TG or low HDLc levels was associated with a reduction in CV disease risk [11, 31]. In addition, a meta-analysis of the effect of fibrates demonstrated a 10% relative risk reduction for MACE and a 13% relative risk reduction for coronary events without a significant increase in serious drug-related adverse events [12]. Although an increased incidence of myopathy was associated with the use of statin plus gemfibrozil, it was not with statin plus fenofibrate therapy [32]. After reviewing our previous findings and the literature, we concluded that fenofibrate plus statin may be an excellent candidate for conferring additive beneficial effects on reduction of residual CV disease risk compared to statin alone therapy. In this study, we will enroll combined dyslipidemia patients (baseline LDLc ≥ 70 mg/dL and non-HDLc ≥ 130 mg/dL in statin-naïve patients, and baseline LDLc ≤ 100 mg/dL and non-HDLc ≥ 100 mg/dL in patients with statin treatment) in order to test our hypothesis.

### The rationale for VH-IVUS-measured percentage NCV as a primary endpoint

The PROSPECT trial revealed that non-culprit lesions account for a substantial proportion of future CV events [5]. Independent predictors for future CV events were a plaque burden ≥ 70%, VH-IVUS-proven TCFA, and a minimum lumen area ≤ 4.0 mm^2^. Similarly, the VIVA study revealed that such predictors were related to a high degree of angiographic diameter stenosis, which may explain why subsequent clinical events were linked to baseline non-culprit lesion severity [33]. As these data prove VH-IVUS as a legitimate in vivo diagnostic modality for delineating the relationship between non-culprit lesions and CV events, we decided to use VH-IVUS to track the quantitative change in NC and the characteristic change in TCFA in both treatment arms.

### Intermediate statin dosage

The incremental benefits associated with higher doses of statins has led to the guidelines recommending the use of high doses of statins in patients with high CV disease risk [2–4, 6, 34]. However, in Asian patients, for the regression of coronary atherosclerotic plaques and reducing MACEs, an intermediate dose of rosuvastatin (10 mg/day) is as effective as high-intensity statins with minimal adverse reactions [35, 36]. The administration of rosuvastatin (10 mg) in the STAFENO trial was based on this rationale.

### Study limitations

The possible limitations of the STAFENO trial are the relatively small study population, although the sample size was calculated based on previous IVUS study, and the relatively short term follow-up.

Therefore, if statin plus fenofibrate shows more beneficial results in terms of changes in plaque volume and characteristics, it will provide the rationale for large-scale randomized clinical trials to demonstrate the efficacy of combined treatment of statin and fenofibrate in non-diabetic CAD patients with combined dyslipidemia.

## Conclusions

We believe that the STAFENO trial will address whether combination statin plus fenofibrate treatment has additive beneficial effects compared to statin alone on the reduction and stabilization of NCV, and the plausible mechanisms of these results, in non-culprit plaques of non-diabetic patients with combined dyslipidemia.

## Trial status

Patient recruitment is ongoing (102/106 subjects, 96.2%) and the completion of the study is estimated to be in December 2020. The latest protocol is version 1.9, last updated on December 17th, 2018.

## Data Availability

The datasets of the current study will be available by the corresponding author upon reasonable request.

## Abbreviations

ACS: Acute coronary syndrome
CAD: Coronary artery disease
CAG: Coronary angiography
CV: Cardiovascular
DC: Dense calcium
FF: Fibrofatty
F: Fibrous
HDLc: High-density lipoprotein cholesterol
LDLc: Low-density lipoprotein cholesterol
MACE: Major adverse cardiac event
NCV: Necrotic core volume
PPAR: Peroxisome proliferator-activated receptor
TCFA: Thin cap fibroatheroma
TG: Triglycerides
QCA: Quantitative coronary angiography
VH-IVUS: Virtual histology-intravascular ultrasound
